# Ras-Association Domain of Sorting Nexin 27 Is Critical for Regulating Expression of GIRK Potassium Channels

**DOI:** 10.1371/journal.pone.0059800

**Published:** 2013-03-25

**Authors:** Bartosz Balana, Laia Bahima, Karthik Bodhinathan, Jaume J. Taura, Natalie M. Taylor, Margaret Y. Nettleton, Francisco Ciruela, Paul A. Slesinger

**Affiliations:** 1 Peptide Biology Laboratories, The Salk Institute for Biological Studies, La Jolla, California, United States of America; 2 Department of Neuroscience, Icahn School of Medicine at Mount Sinai, New York, New York, United States of America; 3 Unitat de Farmacologia, Departament de Patologia i Terapèutica Experimental, University of Barcelona, Barcelona, Spain; Tokyo Metropolitan Institute of Medical Science, Japan

## Abstract

G protein-gated inwardly rectifying potassium (GIRK) channels play an important role in regulating neuronal excitability. Sorting nexin 27b (SNX27b), which reduces surface expression of GIRK channels through a PDZ domain interaction, contains a putative Ras-association (RA) domain with unknown function. Deleting the RA domain in SNX27b (SNX27b-ΔRA) prevents the down-regulation of GIRK2c/GIRK3 channels. Similarly, a point mutation (K305A) in the RA domain disrupts regulation of GIRK2c/GIRK3 channels and reduces H-Ras binding *in vitro*. Finally, the dominant-negative H-Ras (S17N) occludes the SNX27b-dependent decrease in surface expression of GIRK2c/GIRK3 channels. Thus, the presence of a functional RA domain and the interaction with Ras-like G proteins comprise a novel mechanism for modulating SNX27b control of GIRK channel surface expression and cellular excitability.

## Introduction

G protein-gated inwardly rectifying potassium (GIRK or Kir3) channels have been implicated in the pathophysiology of diseases such as epilepsy, Down’s syndrome, and drug addiction [1]. Like other inwardly rectifying K^+^ channels, GIRK channels conduct outward potassium current near the resting membrane potential, thereby hyperpolarizing the cell and decreasing membrane excitability. Stimulation of G protein-coupled receptors (GPCRs) that couple to pertussis toxin-sensitive G proteins (Gαi/o family) activates GIRK channels through G protein Gβγ subunits [2]–[5]. Four mammalian GIRK subunits are expressed in the brain, GIRK1, GIRK2, GIRK3 and to a lesser extent GIRK4. These subunits assemble into heterotetramers of primarily GIRK1/2, GIRK1/3 or GIRK2/3, and homotetramers of GIRK2 [1]. Neurotransmitters, such as GABA, serotonin and adenosine, stimulate their cognate GPCRs and drive the G protein-dependent activation of GIRK channels, leading to inhibition of neuronal activity [6]. Despite their importance in regulating neuronal excitability, little is known about intracellular trafficking pathways and proteins that control expression of GIRK channels.

We recently demonstrated that sorting nexin 27 (SNX27) regulates GIRK channels through a PDZ domain interaction [7], [8]. SNX27 is a member of a large family of sorting nexins (SNXs) that are classified by the presence of lipid-binding phox-homology (PX) domain [9]. Sorting nexins have been identified across different phyla, with 33 different mammalian SNXs [9]. This rapidly expanding family of cytoplasmic proteins is important for regulating protein trafficking [9]. For example, SNX9 participates in the formation of the narrow neck of endocytic vesicles before scission occurs [10], SNX13 controls trafficking of the G protein α_s_ subunit [11], SNX3 is involved in neural development [12], and SNX17 is involved in trafficking of the amyloid precursor protein (APP) implicated in Alzheimer’s disease [13]. SNX27 associates with a variety of signaling proteins, including GIRK channels [7], [8], cytohesin-associated scaffolding protein (CASP) [14], 5-HT4a receptors [15], β2 adrenergic receptors [16], NMDA receptors [17], diacylglycerol kinase zeta (DGKζ) [18] and amyloid precursor protein (APP) [19]. SNX27 protein has been detected in synaptosomes purified from mouse cortex [20], rat hippocampal neurons [7] and lymphocytes [14], and exists in two splice forms, SNX72a and SNX27b [21]. Interestingly, SNX27b may provide a link between drugs of abuse and changes in neuronal excitability [21]. Thus, SNX27 plays an important role in signal transduction and protein trafficking.

SNX27 has three functional domains; PDZ (PSD95/Disc large/Zona occludens), PX (Phox Phagocytic oxidase domain) and RA (Ras-association) domains. The PX domain binds specifically to PI(3)P and targets SNX27 to the early endosomes (EE) [8]. The PDZ domain mediates a direct protein-protein interaction with a Class I PDZ binding motif located in the C-terminal domain of target proteins [7], [8], [15]–[19]. For GIRK channels, the PX and PDZ domains are essential for targeting GIRK3-containing channels to the early endosomes and redirecting away from the plasma membrane, thereby reducing surface expression and producing smaller GIRK currents [7], [8]. However, the role of the RA domain in the SNX27b-dependent regulation of GIRK channel function and neuronal excitability is largely unknown. In general, the RA domain is hypothesized to be involved in facilitating the exchange of GTP for GDP on small Ras-like G proteins [22]. Recently, the carboxyl terminal fragment of SNX13, SNX27 and SNX31, which contains the RA domain, was reported to associate with activated H-Ras in an *in vitro* binding assay [19]. The functional consequence of this interaction remains poorly understood.

In the present study, we investigated the function of the RA domain in the SNX27b-dependent regulation of GIRK channels. We provide new evidence that a functional RA domain is essential for SNX27b control of trafficking and down-regulation of GIRK3-containing channels. These findings provide the first clues into a pathway for regulating SNX27b function in the brain, establishing a possible new link between Ras signaling and neuronal excitability.

## Results and Discussion

**Figure 1:**
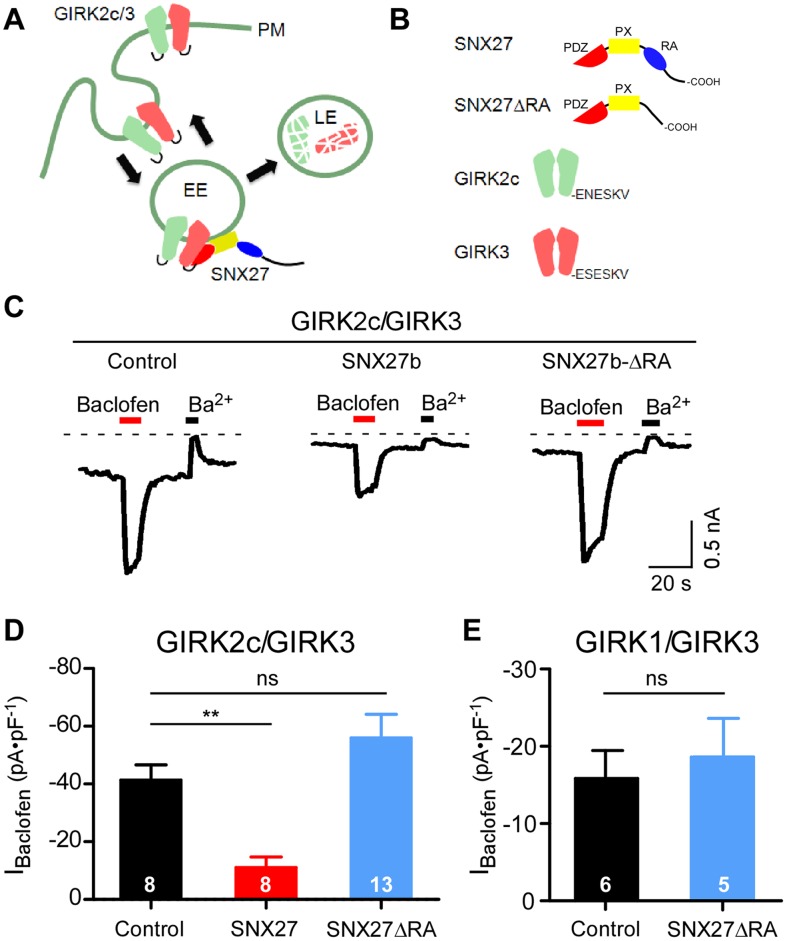
Deletion of RA domain in SNX27b impairs functional regulation of GIRK channels. **A**, Cartoon shows model of GIRK channels regulation by SNX27b. GIRK channels recycle through endosomal compartments. SNX27b associating with GIRK2c/3 channels in the early endosome (EE) reduces plasma membrane expression (PM) by targeting some channels to the late endosome (LE). **B**, SNX27 contains three functional domains; PDZ, PX and RA. GIRK2c and GIRK3 contain a C-terminal PDZ binding motif (-E(S/N)ESKV). **C,** Examples of baclofen-induced (100 µM) and Ba^2+^-sensitive (1 mM Ba^2+^) currents in HEK293T cells transfected with cDNA for GABA_B1a/B2_ receptors, GIRK2c/GIRK3 and either control vector, SNX27b or SNX27b-ΔRA. Agonist-independent basal currents are revealed by inhibition with 1 mM Ba^2+^. **D**, Average baclofen-induced current densities (I_Baclofen_) for control (–41.3±5.2 pA⋅pF^−1^, n = 8), SNX27b (–11.0±3.6 pA⋅pF^−1^, n = 8) and SNX27b- ΔRA (–55.9±8.2 pA⋅pF^−1^, n = 13) with GIRK2c/GIRK3. **E**, Average I_Baclofen_ for control (–15.7±3.6 pA⋅pF^−1^, n = 6) and SNX27b-ΔRA (–18.6±4.9 pA⋅pF^−1^, n = 5) with GIRK1/GIRK3 (**P<0.05, one way ANOVA followed by Bonferroni *post hoc* test; n.s. – not significant).

We have previously observed that overexpression of SNX27b leads to accumulation of GIRK3-containing channels in early endosomes and subsequent reduction in surface expression of GIRK channels (**Figure 1A**) [8]. To investigate the function of the SNX27b RA domain, we first constructed a mutant of SNX27b that lacks the RA domain (SNX27b-ΔRA) (**Figure 1B**). We examined the functional effect of SNX27b by measuring the amplitude of GABA_B_ receptor-activated GIRK currents in HEK293T cells expressing GIRK2c/GIRK3 channels along with GABA_B1_ and GABA_B2_ receptors. Coexpression of SNX27b with GIRK2c/GIRK3 channels significantly reduced the amplitude of GABA_B_R-activated currents (I_Baclofen_) measured with a saturating concentration (100 µM) of the GABA_B_R agonist baclofen (–10 pA/pF vs –41 pA/pF for control, **Figure 1C, 1D**). In contrast to SNX27b, expression of SNX27b-ΔRA did not significantly change I_Baclofen_ (–55 pA/pF) with GIRK2c/3 channels (**Figure 1C, D**). Similarly, SNX27b-ΔRA did not significantly alter I_Baclofen_ for HEK293T cells expressing GIRK1/3 channels (**Figure 1D**), in contrast to previous studies where wild-type SNX27b consistently reduced GIRK1/3 currents [7], [8], [23]. Thus, removal of the RA domain appeared to interfere with the ability of SNX27b to regulate GIRK3-containing channels in HEK293T cells.

**Figure 2:**
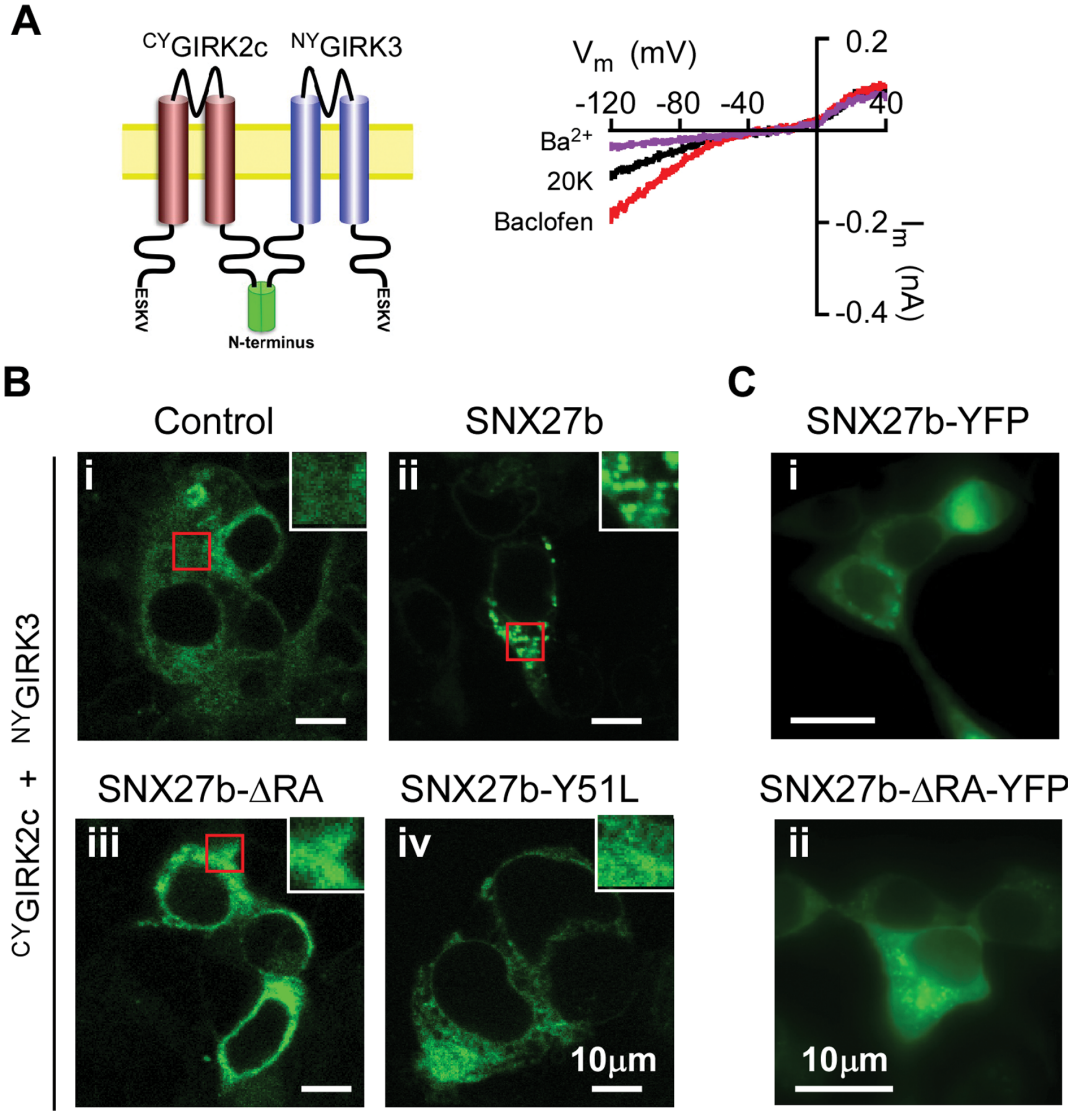
Deletion of RA domain in SNX27b affects localization of GIRK2c/3 channels monitored with BiFC. **A**, *Left*, Schematic shows placement of split YFP on GIRK2c and GIRK3. Note the C-terminal domains are free to interact with other proteins. *Right*, BiFC-tagged GIRK2c/3 channels are functional. Current-voltage plot is shown for ^CY^GIRK2c/^NY^GIRK3 channels. Baclofen (100 µM) activates and Ba^2+^ (1 mM) inhibits inwardly rectifying current. HEK293T cells were transfected with cDNA encoding GABA_B1a_, GABA_B2_ and ^CY^GIRK2c/^NY^GIRK3. Average baclofen-induced current densities were –13.2±6.0 pA⋅pF^−1^ (n = 3) for ^CY^GIRK2c/^NY^GIRK3. **B**, HEK293 cells were co-transfected with ^CY^GIRK2c, ^NY^GIRK3 and either control cDNA (*i*), wild-type SNX27b (*ii*), SNX27b-ΔRA (deletion of Asp272-Trp358) (*iii*) or SNX27b-Y51L (a PDZ mutation) (*iv*). Green fluorescence in images represents molecular recombination of ^CY^GIRK2c/^NY^GIRK3 heterotetramers. Coexpression of wild-type SNX27b induced formation of puncta. By contrast, ^CY^GIRK2c/^NY^GIRK3 fluorescence was diffuse in the cytoplasm for SNX27b-ΔRA and for SNX27b-Y51L, similar to control. Inset shows zoom of boxed area. **C,** SNX27b-ΔRA exhibited a pattern of punctate expression similar that of wild-type SNX27b. YFP was fused to the C-terminus of SNX27b or SNX27b-ΔRA to directly visualize expression. HEK293T cells were transfected with cDNA for SNX27b-YFP and SNX27bΔRA-YFP. Scale bar: 10 µm.

To determine whether SNX27b-ΔRA continued to associate with GIRK2c/3 channels, we next examined the localization of GIRK2c/3 channels in the presence of SNX27b-ΔRA. We used bimolecular fluorescence complementation (BiFC), a novel method for monitoring the expression pattern of GIRK2c/3 heterotetramers (**Figure 2A**) [24]. BiFC involves genetically fusing non-fluorescent, complementary fragments of YFP to two different proteins. When the two proteins interact, as would be the case for two GIRK subunits (e.g. GIRK2c and GIRK3), the two non-fluorescent fragments associate and reconstitute a functional fluorescent protein [24]. ^C^YFP was fused to the N-terminal domain of GIRK2c (^CY^GIRK2c) and ^N^YFP was fused to the N-terminal domain of GIRK3 (^NY^GIRK3) (**Figure 2A**), allowing the unencumbered C-terminal domains of the channels to freely associate with SNX27b. Coexpression of ^CY^GIRK2c and ^NY^GIRK3 led to fluorescently labeled proteins that expressed diffusely in the cytoplasm and functionally coupled with GABA_B_ receptors on the plasma membrane (**Figure 2A,B Control**), similar to a previous study [25]. Expression of SNX27b with^ CY^GIRK2c/^NY^GIRK3 induced puncta of fluorescence in the cytoplasm, typical of endosomal localization (**Figure 2B** **ii**) [7], [8]. Expression of SNX27b-ΔRA, on the other hand, resulted in a diffuse pattern of ^CY^GIRK2c/^NY^GIRK3 fluorescence (**Figure 2B** **iii**), similar to that observed in control cells. Thus, deleting the RA domain appeared to disrupt the interaction of SNX27b-ΔRA with GIRK2c/GIRK3 channels, altering the ability of SNX27b to control the trafficking and surface expression of GIRK channels. This result was unexpected since SNX27b-ΔRA contains intact PDZ and PX domains, which would be expected to associate with the PDZ motif of GIRK2c/3 and endosomal PI(3)P, respectively. Introducing a mutation (Y51L) in the PDZ domain of SNX27b known to disrupt PDZ binding [7] led to a similar diffuse pattern of ^CY^GIRK2c/^NY^GIRK3 fluorescence (**Figure 2B** **iv**), suggesting a potential defect in PDZ binding when the RA domain is deleted. To rule out the possibility that deleting the RA domain simply altered the expression of SNX27b itself, we examined the localization of YFP-tagged SNX27b-ΔRA (SNX27b-ΔRA-YFP). Discrete puncta of green fluorescence typical of endosomal localization were observed in HEK293T cells expressing either SNX27b-YFP or SNX27b-ΔRA-YFP (**Figure 2C**), indicating that SNX27b-ΔRA is expressed and targeted properly.

**Figure 3:**
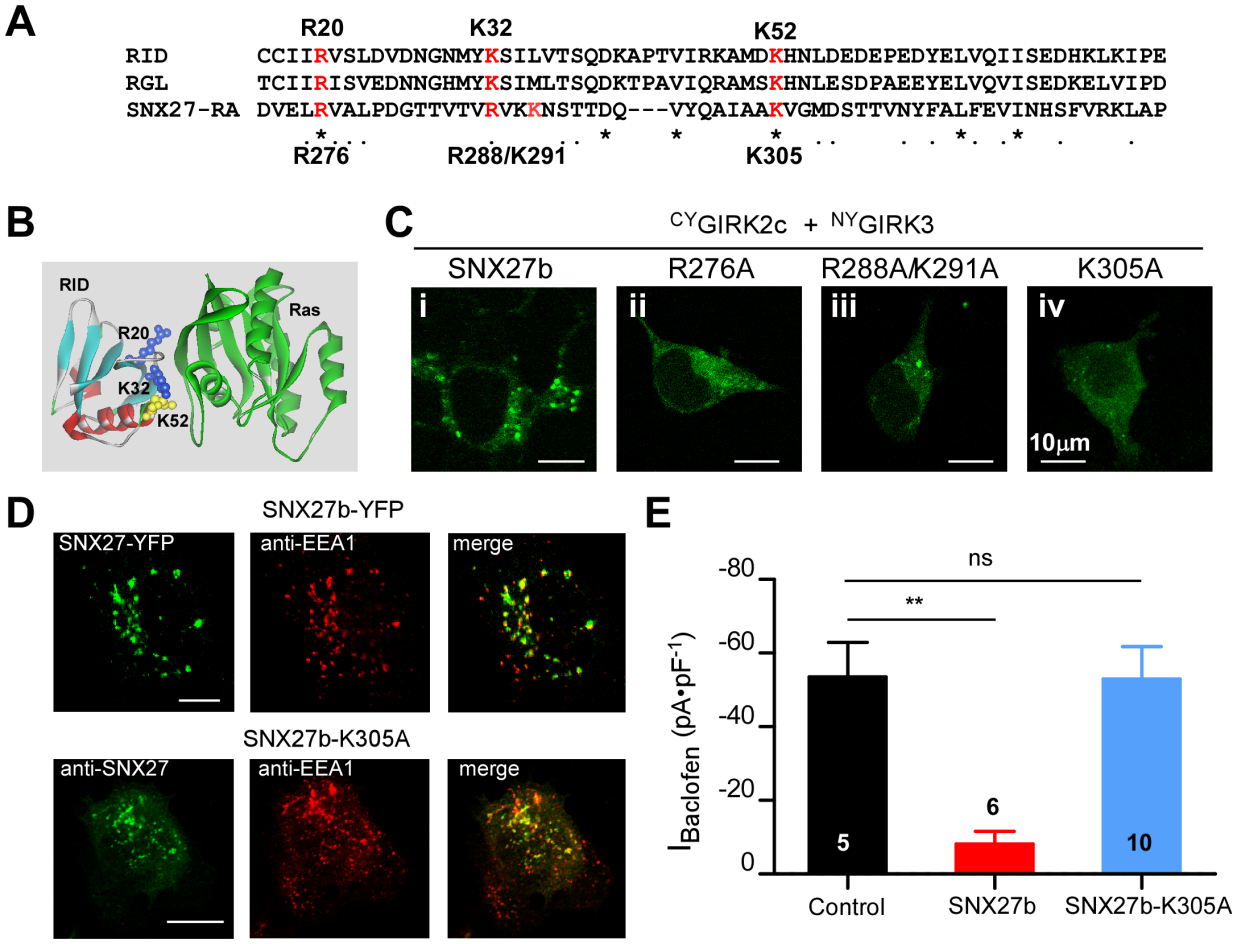
K305A point mutation in SNX27b RA domain disrupts functional regulation of GIRK2c/GIRK3 channels. **A**, Alignment of two different RA domains: a RID (Ras interacting domain) from RalGDS and a RA domain of RGL, with RA domain of SNX27. Residues implicated in Ras binding in Raf and RalGDS domains [28]–[30] are highlighted in red. **B**, High-resolution structure shows R20, K32 and K52 at the binding interface of RID and Ras [26]. **C,** RA domain mutations impair the ability of SNX27b to induce formation of GIRK2c/3 puncta using BiFC. HEK293T cells were co-transfected with cDNA for ^CY^GIRK2c/^NY^GIRK3 and either wild-type SNX27b (**i**), SNX27b-R276A (**ii**), SNX27b-R288A/K291A (**iii**) or SNX27b-K305A (**iv**). Green fluorescence indicates molecular complementation of ^CY^GIRK2c/^NY^GIRK3. Scale bar: 10 µm. **D**, Colocalization of wild-type SNX27b (SNX27b-YFP; green) and SNX27b-K305A (anti-SNX27; green) with an early endosomal marker (anti-EEA1, green). Scale bar: 5 µm. **E**, Average I_Baclofen_ currents for control (–53.4±9.4 pA⋅pF^−1^, n = 5), SNX27b (–8.05±3.44 pA⋅pF^−1^, n = 6) and SNX27b-K305A (–52.9±8.8 pA⋅pF^−1^, n = 10) with GIRK2c/GIRK3 channels.

To investigate the possible mechanism underlying the inability of SNX27b-ΔRA to regulate GIRK channel expression, we examined the effect of selective point mutations in the RA domain. Previously, RA domains have been shown to interact with Ras-like proteins [19], [26]. Examination of a high resolution structure complex of the Ras and the Ras-interacting domain (RID) of Ral or Raf, which is homologous to the RA domain of SNX27b, revealed several basic amino acids (R20, K32, K52) in the RID that electrostatically attract and form hydrogen bonds with acidic residues in Ras-Switch II (e.g. D33) [27]–[30] (**Figure 3B**). We aligned the sequence of SNX27b RA domain with the RID domains of RalGDS and Raf proteins (**Figure 3A**) and identified four basic amino acids in the putative RID of SNX27b that might be involved in binding Ras (R276, R288, K291, K305). We created three mutants, R276A, R288A/K291A and K305A SNX27, which neutralized these basic amino acids, and examined their effects on the expression pattern of ^CY^GIRK2c/^NY^GIRK3 channels using BiFC (**Figure 3C** **)**. Similar to SNX27b-ΔRA, each point mutation appeared to impair the ability of SNX27b to induce punctate fluorescence for ^CY^GIRK2c/^NY^GIRK3 channels (**Figure 3C**). Focusing on SNX27b-K305A, immunostaining revealed partial co-localization with the early endosomal marker, early endosome antigen 1 (EEA1), (**Figure 3D**), similar to wild-type SNX27b [8]. Thus, the PX domain which targets SNX27b to the early endosome appears to be unaffected by the K305A mutation [19]. We then examined the effect of the K305A mutation in SNX27b on GIRK2c/3 channels expressed in HEK293T cells. Similar to SNX27b-ΔRA, SNX27b-K305A did not reduce I_Baclofen_ for GIRK2c/GIRK3 channels (**Figure 3E**).

**Figure 4:**
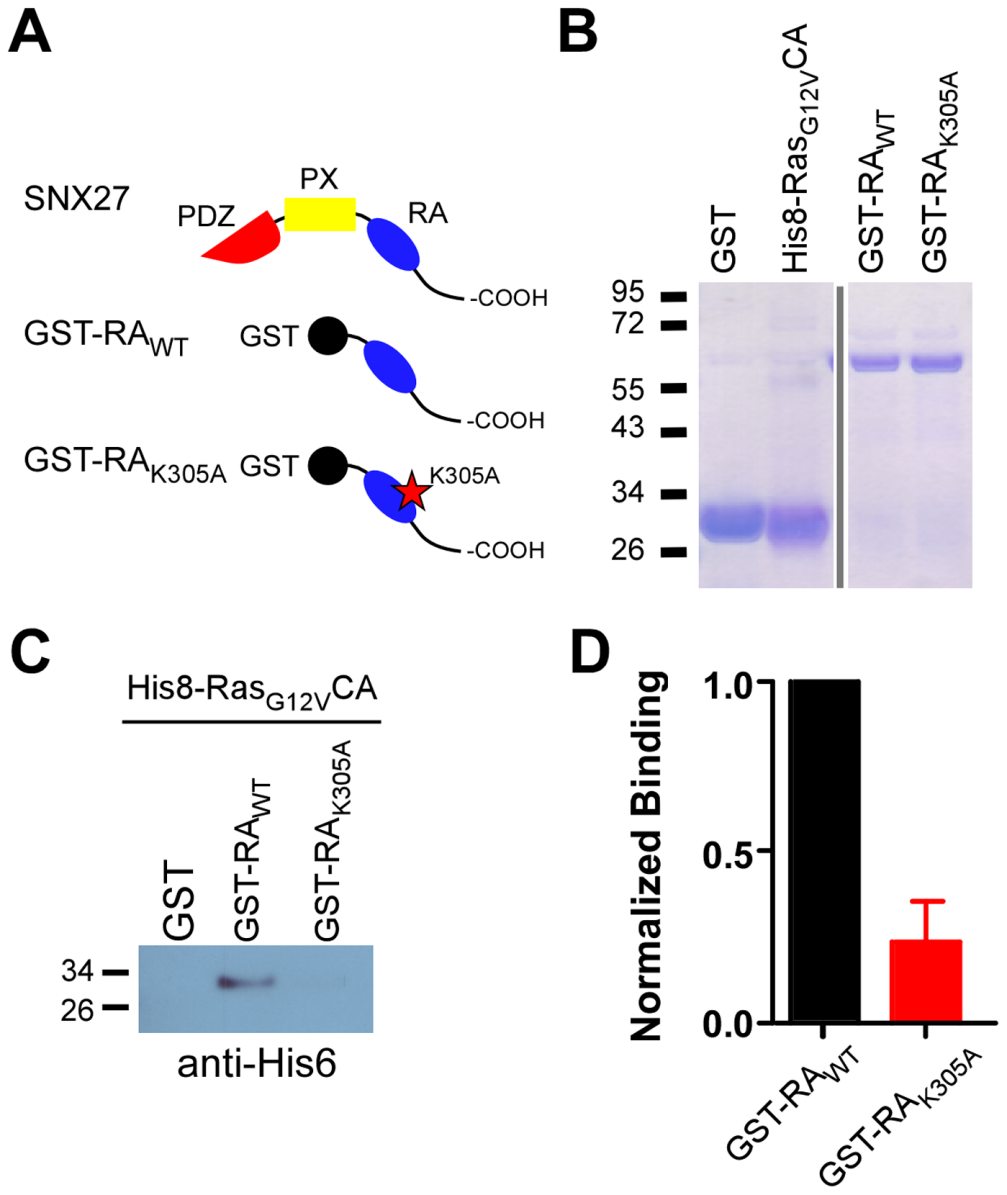
K305A point mutation in SNX27b RA domain disrupts interaction with constitutively active H-Ras (H-Ras_G12V_CA). **A**, Cartoons of SNX27b, GST fused to SNX27b-RA_WT_ or SNX27b-RA_K305A_. **B**, Coomassie stain shows purified GST, His_8_-Ras_G12V_CA, GST-RA_WT_ and GST-RA_K305A_. **C,** Western blot using anti-His_6_ antibody shows His_8_-Ras_G12V_CA binding to GST-RA_WT_ but not to GST-RA_K305A_. **C**, Quantification of pull-down shows consistent decrease in association of GST-RA_K305A_ mutant with His_8_-Ras_G12V_CA (assay repeated 4 times).

To examine whether the K305A mutation in the RA domain interfered with the association of SNX27b with H-Ras, we performed a pull-down experiment using recombinant proteins expressed and purified from *E. coli* (**Figure 4A, 4B**) [19]. A GST fusion protein containing the C-terminal region of SNX27b (Asp272-Tyr526) that includes the RA domain (GST-RA_WT_) pulled down a His_8_-tagged constitutively active form H-Ras, His_8_-H-Ras_G12V_CA (**Figure 4C**). By contrast, GST-RA_K305A_ showed weak or no association with His_8_-H-Ras_G12V_CA (**Figure 4C**). The K305A point mutation consistently reduced by ∼75% the association of the RA domain with H-Ras_G12V_CA (**Figure 4D** **; n = 4**). Taken together, these findings raise the possibility that the interaction of H-Ras with the RA domain is an important step in the SNX27-dependent regulation of GIRK channels.

**Figure 5:**
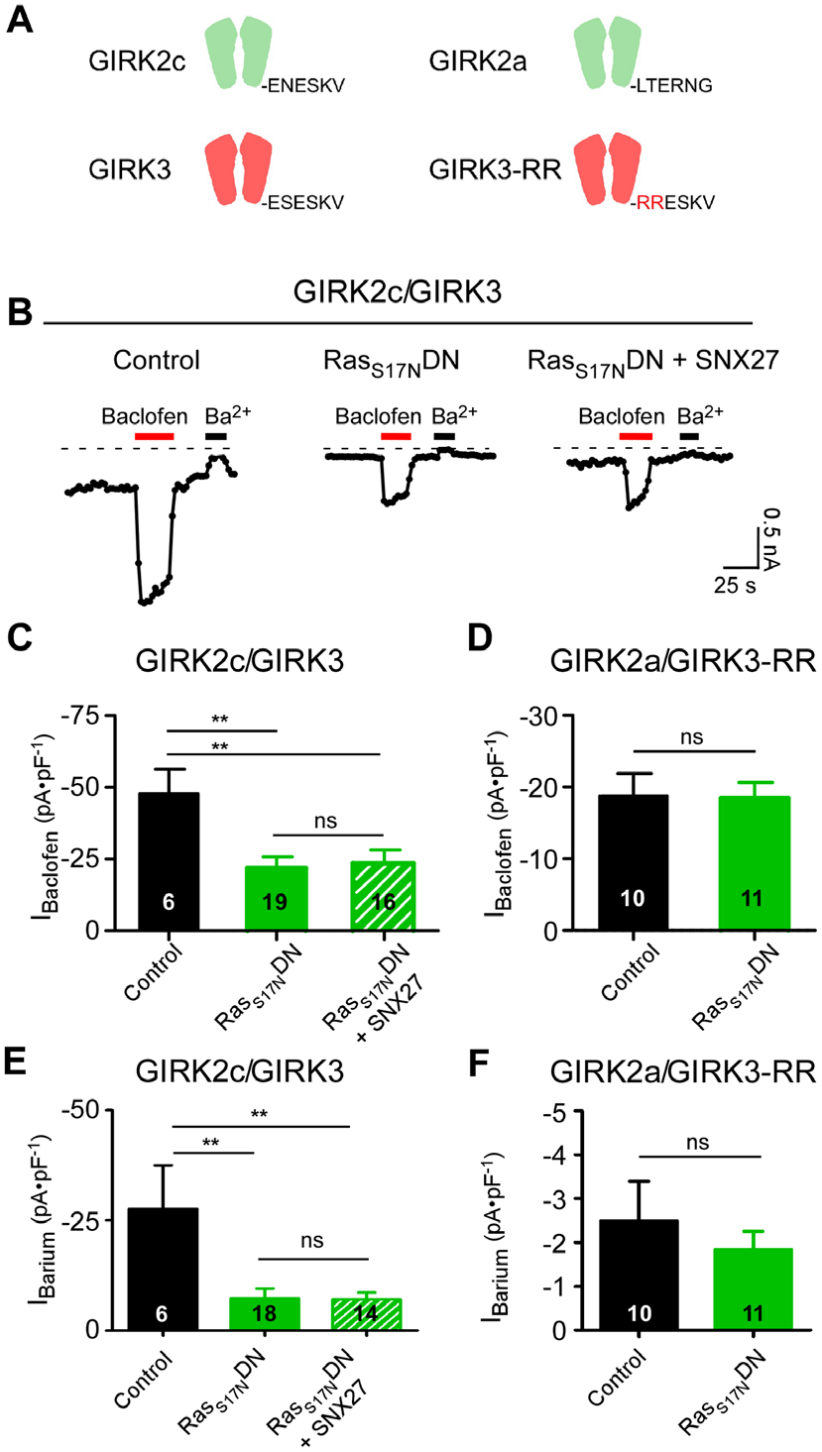
Dominant-negative H-Ras (H-Ras_S17N_DN) prevents SNX27b-dependent down-regulation of GIRK channels. **A**, Schematic shows GIRK2c and GIRK3 with PDZ-binding motif and GIRK2a and GIRK3-RR, which lack motifs that interact with SNX27-PDZ [7]. **B**, Examples of baclofen-induced (100 µM) and Ba^2+^-sensitive (1 mM Ba^2+^) currents in HEK293 cells transfected with cDNA for GABA_B1a/B2_, GIRK2c/GIRK3 and either empty vector (Control), H-Ras_S17N_DN or H-Ras_S17N_DN plus SNX27b. **C**, Bar graph shows average I_Baclofen_ for GIRK2c/GIRK3 alone (–47.7±8.5 pA⋅pF^−1^, n = 6), GIRK2c/GIRK3 and H-Ras_S17N_DN (–22.0±3.8 pA⋅pF^−1^, n = 19) or GIRK2c/GIRK3, SNX27b and H-Ras_S17N_DN (–23.7±4.7 pA⋅pF^−1^, n = 16). **D**, Bar graph shows I_Baclofen_ for control (GIRK2a/GIRK3-RR alone) (–18.8±3.0 pA⋅pF^−1^, n = 10) and GIRK2a/GIRK3-RR plus H-Ras_S17N_DN (–18.6±2.0 pA⋅pF^−1^, n = 11). **E**, Bar graph shows average I_Barium_ for GIRK2c/GIRK3 alone (–27.6±9.8 pA⋅pF^−1^, n = 6), GIRK2c/GIRK3 and H-Ras_S17N_DN (–7.3±2.2 pA⋅pF^−1^, n = 18) or GIRK2c/GIRK3, SNX27b and H-Ras_S17N_DN (–7.1±1.5 pA⋅pF^−1^, n = 14). **F**, Bar graph shows average I_Barium_ for GIRK2a/GIRK3-RR alone (−2.49±0.9 pA⋅pF^−1^, n = 10), GIRK2c/GIRK3 and H-Ras_S17N_DN (−1.84±0.4 pA⋅pF^−1^, n = 11). **P<0.05, one way ANOVA followed by Bonferroni post hoc test; n.s. – not significant.

To explore this hypothesis further, we examined the effect of a dominant-negative (S17N) form of H-Ras (H-Ras_S17N_DN). We hypothesized that H-Ras_S17N_DN would compete for endogenous Ras and possibly impair the ability of SNX27b to regulate GIRK2c/3 channels. In HEK293T cells coexpressing GIRK2c/3 with H-Ras_S17N_DN, I_Baclofen_ was indistinguishable from that in HEK293T cells coexpressing GIRK2c/3, H-Ras_S17N_DN and SNX27b (**Figure 5B, 5C**), suggesting SNX27b regulation of GIRK channels required activated Ras. Interestingly, expression of H-Ras_S17N_DN alone not only reduced I_Baclofen_ (**Figure 5B, 5C**) but also reduced I_Barium_ compared to control cells (**Figure 5E**), suggesting a direct effect of the dominant negative Ras on channel expression perhaps mediated by endogenous SNX27. Indeed, Lauffer et al [16] and Cai et al [17] have demonstrated endogenous expression of SNX27b in HEK293 cells and that knockdown of SNX27 leads to reduced surface expression of β adrenergic receptors [**16**]. Consistent with this, we attempted to measure endogenous SNX27b in the HEK293T cells and detected low levels of protein (data not shown). Thus, SNX27 levels may be tightly regulated where too little or too much SNX27 can interfere with GIRK trafficking to the plasma membrane.

To learn more about the role of Ras proteins, we examined the effect of expressing the constitutively active H-Ras_G12V_CA with GIRK2c/3 in the absence and presence of SNX27. Surprisingly, H-Ras_G12V_CA alone reduced I_Baclofen_ for GIRK2c/3 currents and coexpression with SNX27b did not reduce the current further (**Figure S1A**). However, H-Ras_G12V_CA did not affect the basal I_Barium_ for GIRK2c/3 channels, in contrast to the dominant negative effect of H-Ras_S17N_DN (**Figure S1B**). Furthermore, SNX27b reduced I_Barium_ in the presence of H-Ras_G12V_CA. Taken together, these differences between the dominant-negative and constitutively active H-Ras forms are consistent with a model where the dominant negative Ras effect on GIRK2c/3 involves endogenous SNX27 and the constitutively active form affects other signaling pathways (e.g. GPCR function). We therefore developed an alternative strategy to probe the possible effect of the dominant negative H-Ras by modifying the GIRK channels. Previously, we identified two variants of GIRK2/3 channels that were not regulated by SNX27b; GIRK2a, which lacks a PDZ-binding motif, and GIRK3-RR, which contains two Arginine substitutions in the C-terminal PDZ motif that selectively disrupt PDZ binding [7] (**Figure 5A**). Coexpression of SNX27b with these GIRK2a/GIRK-RR variants did not lead to smaller receptor-activated currents [7]. In contrast to wild-type channels, expression of H-Ras_S17N_DN did not significantly change I_Baclofen_ or I_Barium_ for GIRK2a/GIRK3-RR channels (**Figure 5D,E**). Taken together, these results suggest that Ras_S17N_DN interferes with both endogenously and exogenously expressed SNX27, though additional experiments are needed to probe the role of endogenous SNX27b more explicitly using a knockout strategy.

In summary, the RA domain of SNX27b has an unexpected but important role in modulating SNX27b control of GIRK2c/GIRK3 trafficking. Our findings contribute to an emerging idea that endomembrane-associated Ras-like G proteins are capable of signaling through selective interactions with RA domain-containing proteins, in addition to their well-known plasma membrane signaling pathway [31]. The molecular details on the putative interaction between the RA domain and H-Ras and how it regulates PDZ binding remain unclear. One possibility is the complex of H-Ras with SNX27b recruits sorting machinery required to recycle GIRK channels from the endosome to lysosome or back to the membrane; the destination being dependent on the level of SNX27b present in the cell. Alternatively, the RA domain may regulate PDZ binding through some unknown mechanism. Interestingly, we demonstrated previously that the βB-βC loop of the SNX27-PDZ domain is critical for GIRK3 binding [7], perhaps offering a mechanism for regulating PDZ binding. Nonetheless, the discovery that the RA domain is important for SNX27b function could provide a novel mode for regulating SNX27b function *in vivo*. SNX27b targets other proteins in the brain, including the β2 adrenergic receptor [16], 5-HT4a receptor [15] and NMDAR2C receptor [17]; it will be interesting to see in the future if the RA domain is important for SNX27b regulation of these proteins.

## Methods

### Molecular Biology

For bimolecular fluorescence complementation (BiFC), the N-terminal half (amino acids 1 to 155) of the yellow fluorescent protein (^N^YFP or ^NY^) or the C-terminal half (amino acids 156 to 231) of YFP (^C^YFP or ^CY^) was cloned into XhoI-XbaI sites of pcDNA3.1+ vector (Invitrogen, Carlsbad, CA, USA). The cDNAs encoding GIRK2c or GIRK3 were subcloned into the XbaI site of pcDNA3.1(+) in frame with the sequences for ^N^YFP or ^C^YFP as indicated. The cDNAs for H-Ras (G12V & S17N) were obtained from Missouri S&T cDNA Resource Center (#RASH0000C0, http://www.cdna.org) and subcloned into the pHis8-3 vector downstream of His_8_ tag sequence. A C-terminal fragment containing the RA domain of SNX27b (Asp272-Tyr526) was fused to GST by sub cloning into EcoRI and BamHI sites of pGEX-2T vector (GE Biosciences). Point mutations were introduced by site-directed mutagenesis kit (QuikChange II XL, Agilent) and confirmed by automatic DNA sequencing. SNX27-ΔRA construct has an internal deletion encompassing the RA domain (Asp272-Trp358).

### BiFC Experiments

Human Embryonic Kidney 293 (HEK293) cells were grown in Dulbecco’s modified Eagle’s medium, DMEM (Sigma-Aldrich, St. Louis, MO, USA) supplemented with 1 mM sodium pyruvate, 2 mM L-glutamine, 100 U/ml streptomycin, 100 µg/ml penicillin and 10% (v/v) Fetal Bovine Serum (FBS) at 30 or 37°C and in 5% CO_2_ humidified atmosphere. HEK293 cells were transiently transfected with cDNA using Transfectin (BioRad) or Lipofectamine 2000 (Invitrogen), and incubated at 37°C for 24 h and 30°C for another 24 h, to improve the molecular recombination. Transfected HEK293 cells were fixed in 1% paraformaldehyde solution in phosphate buffered saline (PBS) for 10 min, and washed with phosphate buffered saline (PBS) containing 20 mM glycine to quench any remaining paraformaldehyde. Cells were mounted with Vectashield immuno-fluorescence medium (Vector Laboratories, Peterborough, U.K.) or ProlongGold (Invitrogen, Carlsbad, CA, USA) and imaged with a Leica TCS-SL or Zeiss LSM-710 confocal microscope.

### Immunocytochemistry

HEK293T cells were fixed in 2% paraformaldehyde in PBS for 10 min, washed with PBS, permeabilized with 0.2% Triton-X in PBS, blocked with 1% bovine serum albumin (Jackson ImmunoResearch) in PBS/glycine for 1 h at room temperature. Cells were incubated with mouse anti-early endosome antigen 1 (EEA1; BD Biosciences) antibody (1∶400) or rabbit anti-SNX27 antibody (1∶400) [8] for 1 h, washed and incubated with Alexa 488 or Alexa 647-conjugated secondary antibodies (1∶400; Invitrogen) for 1 h at room temperature. Primary and secondary antibodies were diluted in the blocking solution. Cells on coverslips were mounted as described above.

### Whole-cell Patch-clamp Electrophysiology

HEK293T cells were transfected with 0.2 µg of each channel cDNA of GIRK2c, GIRK2a, GIRK1, GIRK3 and/or GIRK3-RR as indicated for each experiment; and 0.2 µg of each GABA_B1a_ and GABA_B2_ and 0.04 µg EYFP using Lipofectamine 2000 (Life Technologies). Whole-cell patch-clamp recordings were performed 48 h post transfection using borosilicate glass electrodes (Warner Instruments) with 3–5 MΩ resistance when filled with the 130 K intracellular solution [(in mM): 130 KCl, 20 NaCl, 5.46 MgCl_2_, 10 HEPES/Na, pH 7.4, and 5 EGTA/KOH, 2.56 K_2_ATP and 0.3 Li_2_GTP; pH 7.4] Extracellular 20K solution contained (in mM): 156 NaCl, 20 KCl, 2 MgCl_2_, 0.5 CaCl_2_, and 10 HEPES/Na, pH 7.4. Macroscopic membrane currents were recorded using an Axopatch 200B amplifier (Molecular Devices, CA, USA) amplifier, compensated electronically for cell capacitance and series resistance (70–90%), filtered at 2 kHz with an eight-pole Bessel filter, digitized at 5 kHz with a Digidata 1200/1320 A/D interface (Molecular Devices), and stored on a laboratory computer for offline analysis. (±) Baclofen (Sigma-Aldrich, CO, USA) was dissolved in dH_2_O to make a 10 mM stock and diluted directly in the extracellular solution to 100 µM. Data were acquired at room temperature (22–25°C) and analyzed with Clampfit 8.0 (Molecular Devices).

### Protein Biochemistry

Proteins were expressed in BL21DE3 strain of E. coli (NEB, MA, USA). After reaching an optical density OD_600nm_ of 0.6, protein expression was induced with 400 µM IPTG for 4 hours at 28°C. GST and His_8_ proteins were purified using glutathione sepharose beads (GE Healthcare Life Sciences) and Ni-NTA resin (Qiagen, CA, USA), respectively. Protein pull-down assays were performed as described by Ghai et al. [19]. Briefly, purified GST (control) or a GST fused to SNX27-Asp272-Tyr526 (SNX27-RA_D272-Y526_) was mixed with His_8_-H-Ras_G12V_CA for 1 h at room temperature in pull-down buffer (50 mM Tris, 200 mM NaCl, 1 mM DTT, and 1% IGEPAL-CA630, pH 8.0). Protein-protein complexes were pelleted by centrifugation using glutathione sepharose beads, separated on SDS-PAGE gel, transferred to nitrocellulose and exposed to anti-His_6_ antibodies for western detection of His_8_-H-Ras_G12V_CA.

### Statistical and Protein Analyses

Data are presented as mean ± SEM. Statistical differences were examined using unpaired Student’s *t*-test for two groups or one-way ANOVA with Bonferroni *post hoc* test for multiple groups. P<0.05 was considered statistically significant. Protein sequences were first aligned using ClustalV algorithm in LaserGene MegAlign software ver.9.0.4 (DNASTAR Inc., WI, USA) and manually adjusted.

## Supporting Information

Figure S1 **SNX27b-dependent down-regulation of basal GIRK2c/GIRK3 channels is maintained in the presence of constitutively active H-Ras (H-Ras_G12V_CA).** A. Bar graph shows I_Baclofen_ for control (GIRK2c/GIRK3 alone; –66.4±7.2 pA⋅pF^−1^, n = 6) and GIRK2c/GIRK3 plus H-Ras_G12V_CA (–12.9±1.8 pA⋅pF^−1^, n = 5), GIRK2c/GIRK3 plus H-Ras_G12V_CA and SNX27b (–9.75±1.5 pA⋅pF^−1^, n = 7), and GIRK2c/GIRK3 plus SNX27b alone (–12.4±3.5 pA⋅pF^−1^, n = 9). B. Bar graph shows I_Barium_ for control (GIRK2c/GIRK3 alone; –24.6±3.0 pA⋅pF^−1^, n = 6) and GIRK2c/GIRK3 plus H-Ras_G12V_CA (–23.3±7.1 pA⋅pF^−1^, n = 5), GIRK2c/GIRK3 plus H-Ras_G12V_CA and SNX27b (–5.79±0.9 pA⋅pF^−1^, n = 7), and GIRK2c/GIRK3 plus SNX27b (–4.84±1.5 pA⋅pF^−1^, n = 8). **P<0.05, one way ANOVA followed by Bonferroni post hoc test; n.s. – not significant.
(TIF)
