# Supplementary figures and images for: Ras-Association Domain of Sorting Nexin 27 Is Critical for Regulating Expression of GIRK Potassium Channels
Source: PLoS One. 2013 Mar 25;8(3):e59800. doi: 10.1371/journal.pone.0059800 (PMC3607560; doi:10.1371/journal.pone.0059800)

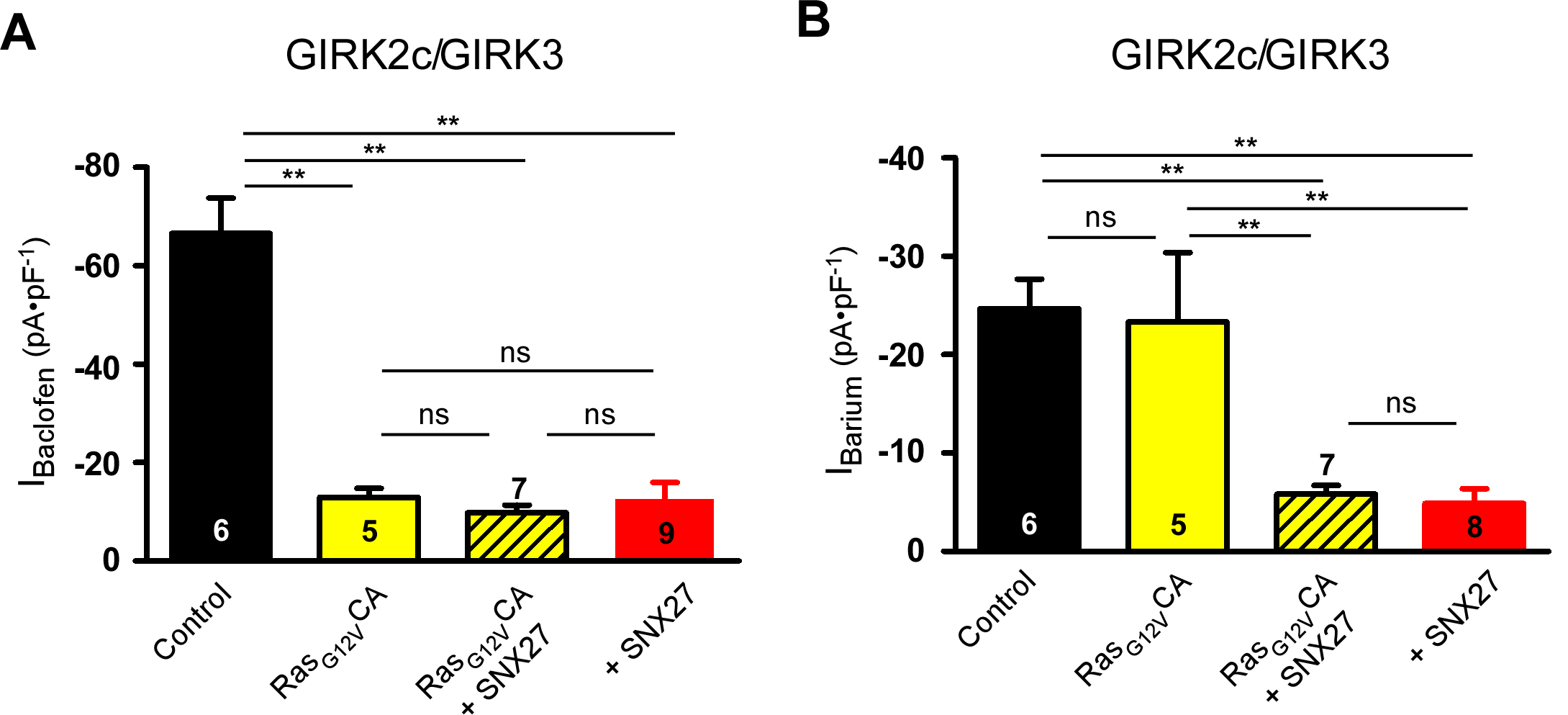

Supplement: Figure S1 — SNX27b-dependent down-regulation of basal GIRK2c/GIRK3 channels is maintained in the presence of constitutively active H-Ras (H-RasG12VCA). A. Bar graph shows IBaclofen for control (GIRK2c/GIRK3 alone; –66.4±7.2 pA⋅pF−1, n = 6) and GIRK2c/GIRK3 plus H-RasG12VCA (–12.9±1.8 pA⋅pF−1, n = 5), GIRK2c/GIRK3 plus H-RasG12VCA and SNX27b (–9.75±1.5 pA⋅pF−1, n = 7), and GIRK2c/GIRK3 plus SNX27b alone (–12.4±3.5 pA⋅pF−1, n = 9). B. Bar graph shows IBarium for control (GIRK2c/GIRK3 alone; –24.6±3.0 pA⋅pF−1, n = 6) and GIRK2c/GIRK3 plus H-RasG12VCA (–23.3±7.1 pA⋅pF−1, n = 5), GIRK2c/GIRK3 plus H-RasG12VCA and SNX27b (–5.79±0.9 pA⋅pF−1, n = 7), and GIRK2c/GIRK3 plus SNX27b (–4.84±1.5 pA⋅pF−1, n = 8). **P<0.05, one way ANOVA followed by Bonferroni post hoc test; n.s. – not significant. (TIF) [file pone.0059800.s001.tif]
